# Supplementary material for: Improving split‐HaloTag through computational protein engineering
Source: Protein Sci. 2025 Apr 18;34(5):e70123. doi: 10.1002/pro.70123 (PMC12006747; doi:10.1002/pro.70123)
Supplement: Supplementary file 1 — Data S1. Supporting Information [file PRO-34-e70123-s001.zip › cpHd2_SI_rev.docx]

Supplementary Materials for

Improving Split-HaloTag through

Computational Protein Engineering

Jonas Wilhelm^1^, Lennart Nickel^1^, Yin-Hsi Lin^1^, Julien Hiblot^1^, Kai Johnsson^1,2^

^1^ Department of Chemical Biology, Max Planck Institute for Medical Research, Jahnstrasse 29, 69120 Heidelberg, Germany.

^2^ Institute of Chemical Sciences and Engineering (ISIC), École Polytechnique Fédérale de Lausanne (EPFL), 1015 Lausanne, Switzerland

# This PDF file includes:

Supplementary figures S1 to S8

Supplementary tables S1 to S3

Protein sequences

Comments on the choice of models for kinetic data analysis

References

# Supplementary Figures

**Fig. S1. Labeling kinetics of cpHalo∆ point mutants.**

Fluorescence polarization labeling kinetics of cpHalo∆ point mutants (100 nM) with TMR-CA (20 nM) in presence or absence of Hpep3 (6.25 μM). Second-order reaction models or linear models (if reactions did not plateau) were fitted to the data to determine labeling rates.

Fig. S2. Melting temperatures and relative labeling speeds of screened cpHalo∆ point mutants.

Melting temperatures were determined by nanoDSF, labeling rates via fluorescence polarization (see Fig. S1) at non-saturating Hpep concentrations.

**Fig. S3.** **Labeling kinetics of cpHalo∆ point mutants after His-tag removal.**

Fluorescence polarization labeling kinetics of cpHalo∆ point mutants (100 nM) after His-tag removal via TEVp digestion, measured with TMR-CA (20 nM) in presence or absence of Hpep3 (6.25 μM). Second-order reaction models or linear models (if reactions did not plateau) were fitted to the data to determine labeling rates.

Fig. S4. Labeling kinetics of cpHalo∆ variants with designed circular permutation linkers.

Fluorescence polarization labeling kinetics of cpHalo∆ variants (100 nM) with TMR-CA (20 nM) in presence or absence of Hpep3 (6.25 μM). Second-order reaction models or linear models (if reactions did not plateau) were fitted to the data to determine labeling rates.

Fig. S5. Melting temperatures and relative labeling speeds of screened cpHalo∆ variants with designed circular permutation linkers.

Melting temperatures were determined by nanoDSF, labeling rates via fluorescence polarization (see Fig. S4) at non-saturating Hpep concentrations.


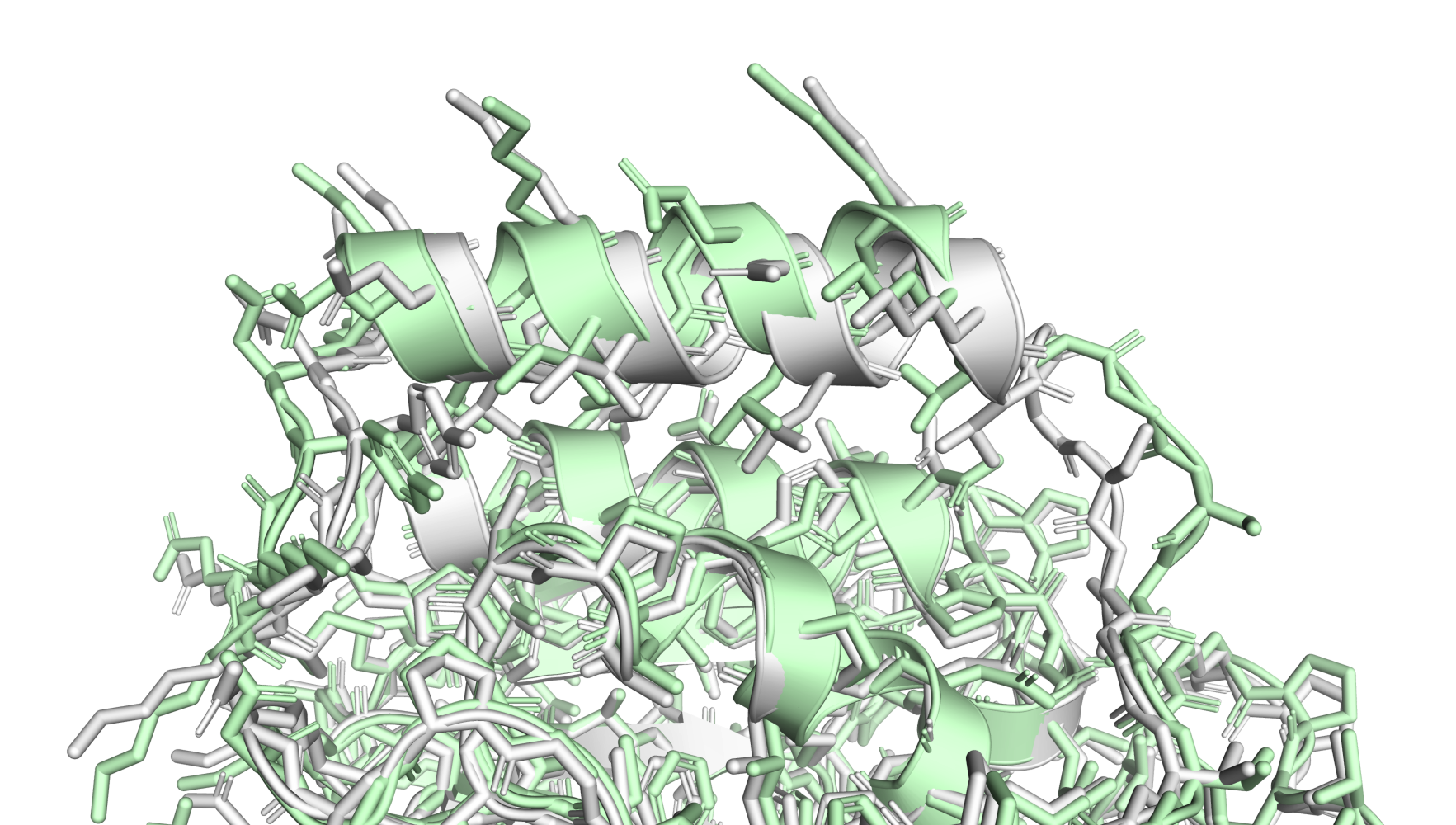


Fig. S6. Overlay of the design model with the final circular permutation linker and the AlphaFold 3 predicted structure.

The design model (with linker_04) is shown in grey, the AlphaFold 3 predicted structure in green (RMSD 0.530 Å).

**Fig. S7.** **Labeling kinetics of cpHalo∆ variants featuring combinations of mutations / CP-linker exchange.**

Fluorescence polarization labeling kinetics of cpHalo∆ variants (100 nM) after His-tag removal via TEVp digestion, measured with TMR-CA (20 nM) in presence or absence of Hpep3 (6.25 μM). Second-order reaction models were fitted to the data to determine labeling rates.

Fig. S8. cpHalo∆2 labeling kinetics at varying Hpep1-8 concentrations.

Fluorescence polarization labeling kinetics of cpHalo∆ variants (10 nM) with TMR-CA (2 nM) at varying concentrations of Hpep1-8. Second-order reaction models or linear models (if reactions did not plateau) were fitted to the data to determine labeling rates.


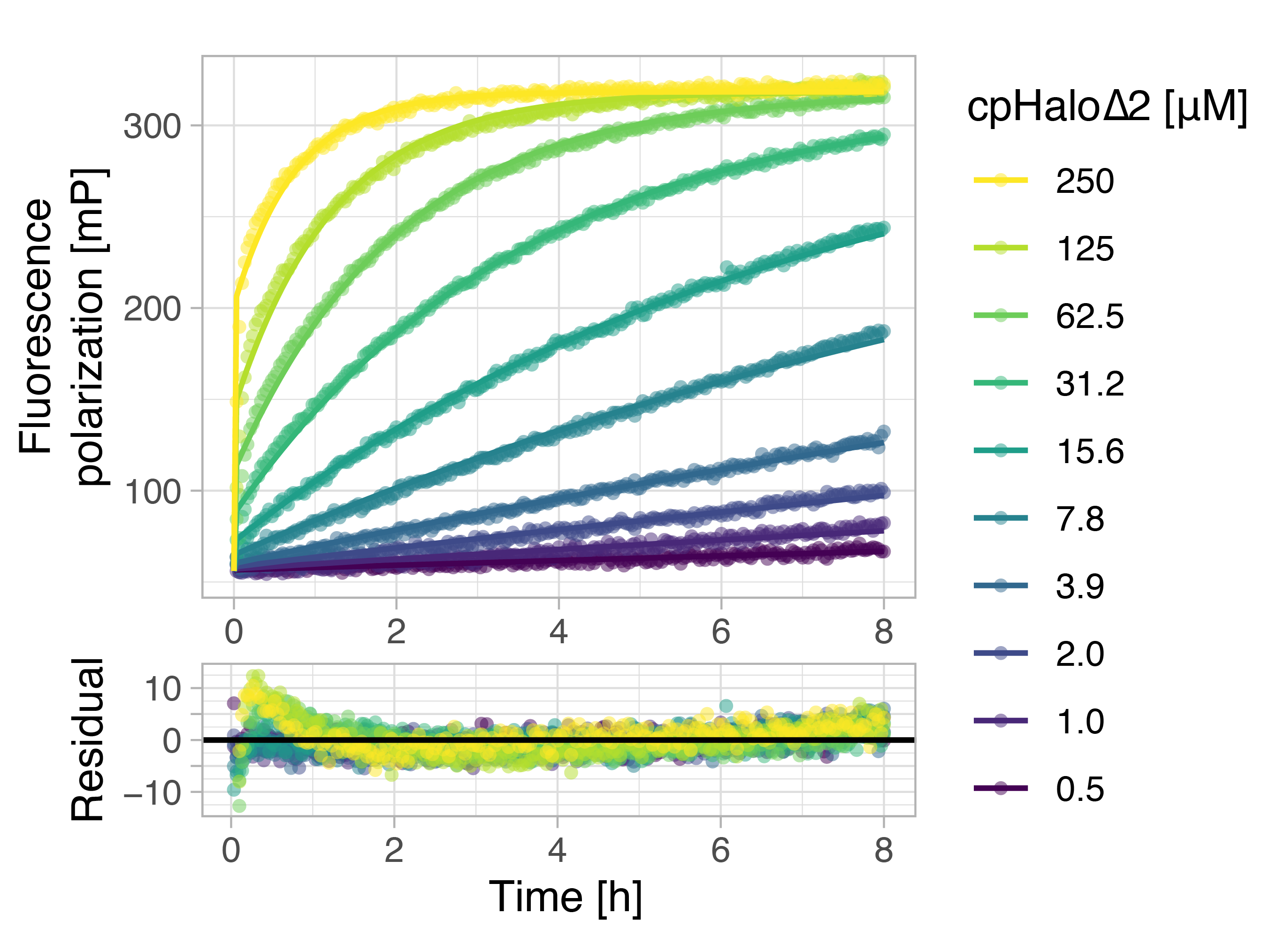


Fig. S9. Background labeling of cpHalo∆2 in absence of Hpep

Fluorescence polarization labeling kinetics of cpHalo∆2 at various concentrations with TMR-CA (50 nM). A two-step reaction model was fitted to the data globally to determine the kinetic parameters of the background labeling reaction (see table S3). Residuals of the fit are shown below.


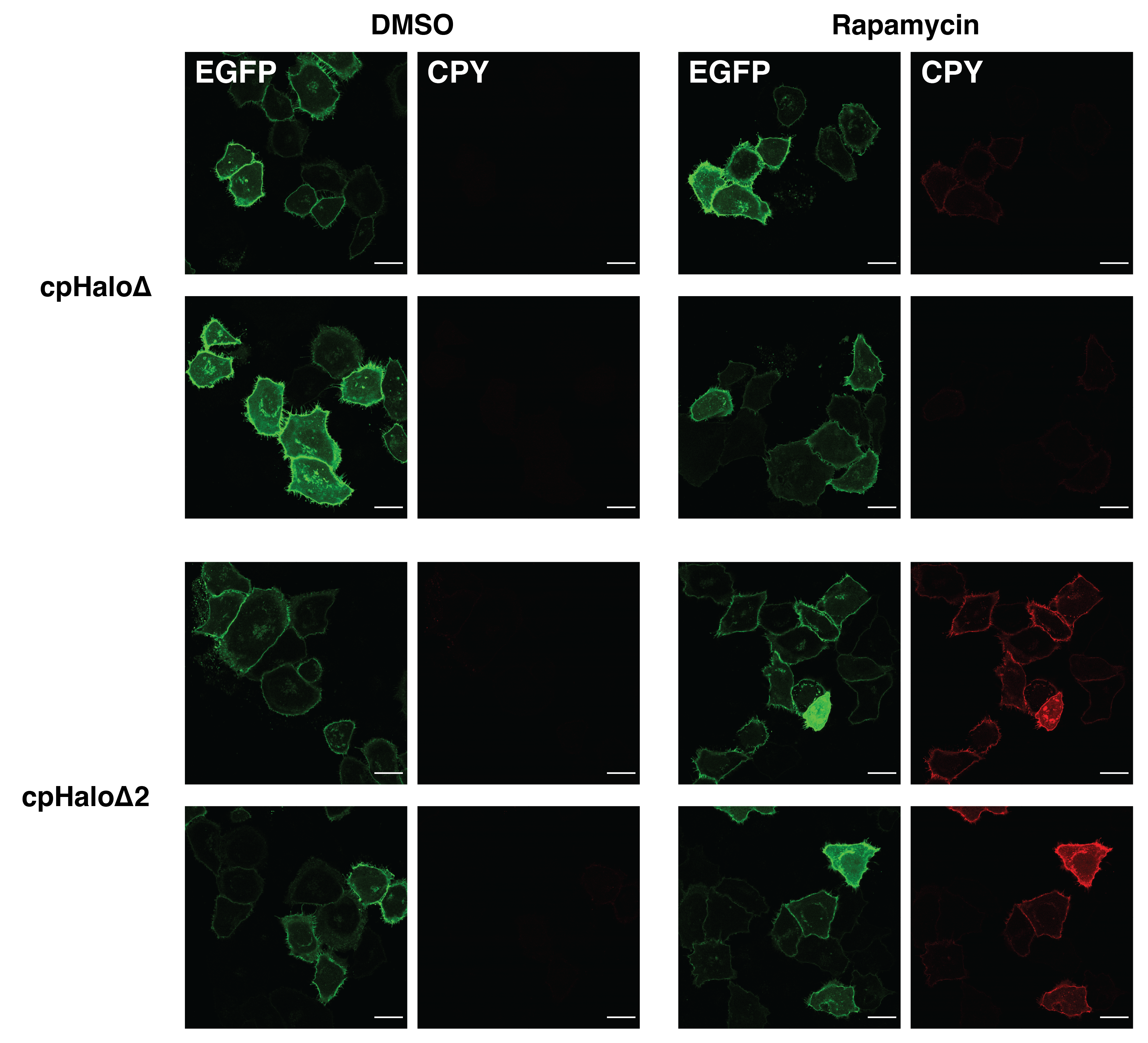


Fig. S10. Additional fluorescence micrographs of rapamycin-dependent labeling of FKBP/FRB split-HaloTag fusions in HeLa cells.

Additional confocal fluorescence micrographs of HeLa cells co-expressing Lyn11-EGFP-cpHalo∆/cpHalo∆2-(GGS)_9_-FKBP and Hpep3-(GGS)_3_-FRB-mScarlet. Cells were treated with CPY-CA (10 nM, 30 min) in presence or absence of rapamycin (100 nM). Scale bars are 25 μm.

Fig. S11. Expression levels of FKBP/FRB split-HaloTag fusions in HeLa cells measured by flow cytometry.

(A, B) Flow cytometry analysis of Lyn11-EGFP-cpHalo∆/cpHalo∆2-(GGS)_9_-FKBP and Hpep3-(GGS)_3_-FRB-mScarlet expression levels in HeLa cells. Data correspond to the same cells analyzed in Fig. 2D.

Fig. S12. Rapamycin-dependent labeling of FKBP/FRB split-HaloTag fusions with different fluorescent HaloTag substrates measured by flow cytometry.

Flow cytometry analysis of HeLa cells co-expressing Lyn11-EGFP-cpHalo∆/cpHalo∆2-(GGS)_9_-FKBP and Hpep3-(GGS)_3_-FRB-mScarlet labeled with different fluorescent HaloTag substrates (10 nM, 30 min) in the presence or absence of rapamycin (RAPA, 100 nM). cpHalo∆2 shows increased labeling ratios in presence of rapamycin (10.6-fold higher on average) and a larger difference in labeling with or without rapamycin. For results with CPY-CA see Fig. 2D. SiR: silicon rhodamine.

# Supplementary tables

Table S1. Apparent second-order rate constants (k_app_) of cpHalo∆ and cpHalo∆2 at saturating concentrations of Hpep3.

Labeling rates were determined with TMR-CA at 37 ºC with a 20-minute pre-incubation before starting the reaction.

| **Variant** | **k_app_** | **95% confidence interval** |
| --- | --- | --- |
| cpHalo∆ | 0.0978 · 10^6^ | (0.0957 – 0.0999) · 10^6^ |
| cpHalo∆2 | 1.61 · 10^6^ | (1.57 – 1.64) · 10^6^ |

**Table S2. EC_50_ values for the cpHalo∆2 labeling reaction with Hpep variants 1-8.**

EC_50_ values were determined with TMR-CA at 37 ºC with a 20-minute pre-incubation before starting the reaction.

| **Variant** | **Sequence** | **EC_50_ (μM)** | **95% confidence interval** |
| --- | --- | --- | --- |
| Hpep1 | ARETFQAFRT | 2428 | (2077 – 2862) |
| Hpep2 | AREMFQAFRT | 283 | (262 – 306) |
| Hpep3 | SKRDAREMFQAFRT | 35.9 | (31.0 – 41.9) |
| Hpep4 | WKEEVIKAFKLFRD | 1.37 | (1.08 – 1.73) |
| Hpep5 | WREEVRKAFKLFRQ | 0.930 | (0.806 – 1.068) |
| Hpep6 | WRETFQLFRT | 0.796 | (0.710 – 0.889) |
| Hpep7 | WREMFRLFRT | 0.130 | (0.105 – 0.159) |
| Hpep8 | WKRDWREMFRLFRT | 0.0434 | (0.0383 – 0.0491) |

Table S3. Kinetic parameters of cpHalo∆2 background labeling in absence of Hpep.

Parameters were determined with TMR-CA at 37 ºC with a 20-minute pre-incubation before starting the reaction.

| **Parameter** | **Value** | **95% confidence interval** |
| --- | --- | --- |
| K_D_ | 201 μM | (192 – 210) μM |
| k_2_ | 6.55 · 10^-4^ s^-1^ | (6.28 – 6.82) · 10^-4^ s^-1^ |
| k_app_ | 3.26 M^-1^s^-1^ | (3.14 – 3.39) M^-1^s^-1^ |

# Protein sequences

>HaloTag (as reference for numbering of mutations)

MAEIGTGFPFDPHYVEVLGERMHYVDVGPRDGTPVLFLHGNPTSSYVWRNIIPHVAPTHRCIAPDLIGMGKSDKPDLGYFFDDHVRFMDAFIEALGLEEVVLVIHDWGSALGFHWAKRNPERVKGIAFMEFIRPIPTWDEWPEFARETFQAFRTTDVGRKLIIDQNVFIEGTLPMGVVRPLTEVEMDHYREPFLNPVDREPLWRFPNELPIAGEPANIVALVEEYMDWLHQSPVPKLLFWGTPGVLIPPAEAARLAKSLPNCKAVDIGPGLNLLQEDNPDLIGSEIARWLSTLEISG

>cpHalo∆

MHHHHHHHHHHENLYFQGDVGRKLIIDQNVFIEGTLPMGVVRPLTEVEMDHYREPFLNPVDREPLWRFPNELPIAGEPANIVALVEEYMDWLHQSPVPKLLFWGTPGVLIPPAEAARLAKSLPNCKAVDIGPGLNLLQEDNPDLIGSEIARWLSTLEIGGTGGSGGTGGSGGSIGTGFPFDPHYVEVLGERMHYVDVGPRDGTPVLFLHGNPTSSYVWRNIIPHVAPTHRCIAPDLIGMGKSDKPDLGYFFDDHVRFMDAFIEALGLEEVVLVIHDWGSALGFHWAKRNPERVKGIAFMEFIRPIPTWDEW

His-tag, TEVp cleavage site, circular permutation linker

Designed circular permutation linkers

>linker_01

RSDDPRKTQTIASKISRDLNGS

>linker_02

KGGTKRDADKAVRDTLLSLNGQ

>linker_03

GGAPRDEALKKIEKAKRDTGDQ

>linker_04 (final linker)

KSKYDRDQILKIIAELEKKTGGS

>linker_05

KSKYDKRQIRDIADKIAKDNNHQ

>linker_06

QSKYPPEWLEKVIRELLKRKNGR

>linker_07

GADDKTKIEKILEEIKRRWQGR

>linker_08

GTSDPRNQEIAKKLARDASTVP

>linker_09

NGADKEQIDRAIEKAKRDLNNQ

>linker_10

KGASDRDEAKKLADDIRKKKGDQ

>linker_11

NSNGHRDELEKILQTIRKQNNDI

>linker_12

LKDERQRDKALEIADRADKYPTS

>cpHalo∆2

MHHHHHHHHHHENLYFQGDVGRKLIIDQNVFIEGTLPMGVVRPLTEEEMDHYREPFLNPKDREPLWRFPNELPIAGEPANIVALVEEYMDWLHQSPVPKLLFWGTPGVLIPPAEAARLAKSLPNCKAVDIGPGLNLLQEDNPDLIGSEIARWLSTLEIKSKYDRDQILKIIAELEKKTGGSIGTGFPFDPHYVEVLGSRMHYVDVGPRDGTPVLFLHGNPTSSYVWRNIIPHVAPTHRCIAPDLIGMGKSDKPDLGYFFDDHVRFMDAFIEALGLEEVVLVIHDWGSALGFHWAKRHPERVKGIAFMEFIRPIPTWDEW

His-tag, TEVp cleavage site, circular permutation linker, mutations relative to cpHalo∆

Hpep sequences

>Hpep1

ARETFQAFRT

>Hpep2

AREMFQAFRT

>Hpep3

SKRDAREMFQAFRT

>Hpep4

WKEEVIKAFKLFRD

>Hpep5

WREEVRKAFKLFRQ

>Hpep6

WRETFQLFRT

>Hpep7

WREMFRLFRT

>Hpep8

WKRDWREMFRLFRT

Lyn11-EGFP-cpHalo∆-(GGS)_9_-FKBP-P2A-Hpep3-(GGS)_3_-FRB-mScarlet

MGCIKSKGKDSAGADSAGSAGMVSKGEELFTGVVPILVELDGDVNGHKFSVSGEGEGDATYGKLTLKFICTTGKLPVPWPTLVTTLTYGVQCFSRYPDHMKQHDFFKSAMPEGYVQERTIFFKDDGNYKTRAEVKFEGDTLVNRIELKGIDFKEDGNILGHKLEYNYNSHNVYIMADKQKNGIKVNFKIRHNIEDGSVQLADHYQQNTPIGDGPVLLPDNHYLSTQSALSKDPNEKRDHMVLLEFVTAAGITLGMDELYKGSGGSGDVGRKLIIDQNVFIEGTLPMGVVRPLTEVEMDHYREPFLNPVDREPLWRFPNELPIAGEPANIVALVEEYMDWLHQSPVPKLLFWGTPGVLIPPAEAARLAKSLPNCKAVDIGPGLNLLQEDNPDLIGSEIARWLSTLEIGGTGGSGGTGGSGGSIGTGFPFDPHYVEVLGERMHYVDVGPRDGTPVLFLHGNPTSSYVWRNIIPHVAPTHRCIAPDLIGMGKSDKPDLGYFFDDHVRFMDAFIEALGLEEVVLVIHDWGSALGFHWAKRNPERVKGIAFMEFIRPIPTWDEWGSGGTGGSGGSGGTGGSGGSGGTGGSGMGVQVETISPGDGRTFPKRGQTCVVHYTGMLEDGKKFDSSRDRNKPFKFMLGKQEVIRGWEEGVAQMSVGQRAKLTISPDYAYGATGHPGIIPPHATLVFDVELLKLEGSGATNFSLLKQAGDVEENPGPGGSSKRDAREMFQAFRTGGSGGTGGSAILWHEMWHEGLEEASRLYFGERNVKGMFEVLEPLHAMMERGPQTLKETSFNQAYGRDLMEAQEWCRKYMKSGNVKDLLQAWDLYYHVFRRISKGSGVSKGEAVIKEFMRFKVHMEGSMNGHEFEIEGEGEGRPYEGTQTAKLKVTKGGPLPFSWDILSPQFMYGSRAFTKHPADIPDYYKQSFPEGFKWERVMNFEDGGAVTVTQDTSLEDGTLIYKVKLRGTNFPPDGPVMQKKTMGWEASTERLYPEDGVLKGDIKMALRLKDGGRYLADFKTTYKAKKPVQMPGAYNVDRKLDITSHNEDYTVVEQYERSEGRHSTG

Lyn11, EGFP, cpHalo∆, FKBP, P2A, Hep3, FRB, mScarlet

Lyn11-EGFP-cpHalo∆2-(GGS)_9_-FKBP-P2A-Hpep3-(GGS)_3_-FRB-mScarlet

MGCIKSKGKDSAGADSAGSAGMVSKGEELFTGVVPILVELDGDVNGHKFSVSGEGEGDATYGKLTLKFICTTGKLPVPWPTLVTTLTYGVQCFSRYPDHMKQHDFFKSAMPEGYVQERTIFFKDDGNYKTRAEVKFEGDTLVNRIELKGIDFKEDGNILGHKLEYNYNSHNVYIMADKQKNGIKVNFKIRHNIEDGSVQLADHYQQNTPIGDGPVLLPDNHYLSTQSALSKDPNEKRDHMVLLEFVTAAGITLGMDELYKGSGGSGDVGRKLIIDQNVFIEGTLPMGVVRPLTEEEMDHYREPFLNPKDREPLWRFPNELPIAGEPANIVALVEEYMDWLHQSPVPKLLFWGTPGVLIPPAEAARLAKSLPNCKAVDIGPGLNLLQEDNPDLIGSEIARWLSTLEIKSKYDRDQILKIIAELEKKTGGSIGTGFPFDPHYVEVLGSRMHYVDVGPRDGTPVLFLHGNPTSSYVWRNIIPHVAPTHRCIAPDLIGMGKSDKPDLGYFFDDHVRFMDAFIEALGLEEVVLVIHDWGSALGFHWAKRHPERVKGIAFMEFIRPIPTWDEWGSGGTGGSGGSGGTGGSGGSGGTGGSGMGVQVETISPGDGRTFPKRGQTCVVHYTGMLEDGKKFDSSRDRNKPFKFMLGKQEVIRGWEEGVAQMSVGQRAKLTISPDYAYGATGHPGIIPPHATLVFDVELLKLEGSGATNFSLLKQAGDVEENPGPGGSSKRDAREMFQAFRTGGSGGTGGSAILWHEMWHEGLEEASRLYFGERNVKGMFEVLEPLHAMMERGPQTLKETSFNQAYGRDLMEAQEWCRKYMKSGNVKDLLQAWDLYYHVFRRISKGSGVSKGEAVIKEFMRFKVHMEGSMNGHEFEIEGEGEGRPYEGTQTAKLKVTKGGPLPFSWDILSPQFMYGSRAFTKHPADIPDYYKQSFPEGFKWERVMNFEDGGAVTVTQDTSLEDGTLIYKVKLRGTNFPPDGPVMQKKTMGWEASTERLYPEDGVLKGDIKMALRLKDGGRYLADFKTTYKAKKPVQMPGAYNVDRKLDITSHNEDYTVVEQYERSEGRHSTG

Lyn11, EGFP, cpHalo∆, FKBP, P2A, Hep3, FRB, mScarlet

# Comments on the choice of models for kinetic data analysis

Most kinetic data in this manuscript were analyzed using a one-step, second-order reaction model, yielding a single apparent second-order rate constant ($k_{app}$, equation 1).

$$\begin{aligned} P+S\underset{\to}{k_{app}}PS\#\left( 1 \right) \end{aligned}$$

However, the kinetics of HaloTag labeling, and likely those of split-HaloTag labeling, are more accurately described by a two-step reaction model that distinguishes substrate binding/unbinding and covalent bond formation with three kinetic constants (k_1_, k_-1_, and k_2_; equations 2–3)^1^.

$$\begin{aligned} P+S\begin{matrix} k_{1} \\ \underset{\leftrightarrow}{} {PS}^{*} \\ k_{-1} \end{matrix}\#\left( 2 \right) \end{aligned}$$

$$\begin{aligned} {PS}^{*}\underset{\to}{k_{2}} PS\#\left( 3 \right) \end{aligned}$$

Nevertheless, the simplified model (equation 1) is justified under certain experimental conditions. At concentrations below the K_D_ (327 nM for HaloTag with TMR-CA^1^ and likely higher for complemented split-HaloTag), the two models converge with minimal deviations. This simplification enables direct fitting of $k_{app}$ values from a single time course experiment recorded at relatively low protein concentrations. In contrast, confidently fitting the two-step model would require data recorded at a range of protein concentrations near or exceeding the K_D_. Such measurements would require more complex setups (e.g., a stopped flow device) due to the fast kinetics at higher concentrations and would also demand substantial quantities of protein rendering them unsuitable for screening.

To investigate the background labeling kinetics of cpHalo∆2, high protein concentrations had to be used in order to reach a plateau within 8 hours, avoiding evaporation issues during longer measurements. These concentrations seemed to exceed the K_D_ of the protein-substrate interaction, leading to a two-phasic behavior in the traces (binding phase and covalent reaction phase, Fig. S9). Hence, we fitted the two-step model to these data and derived the apparent second-order rate constant under non-saturating conditions using equation 4.

$$\begin{aligned} k_{app}=k_{1}\cdot\frac{k_{2}}{k_{2}+k_{-1}}\#\left( 4 \right) \end{aligned}$$

# References

1. Wilhelm J, Kühn S, Tarnawski M, Gotthard G, Tünnermann J, Tänzer T, Karpenko J, Mertes N, Xue L, Uhrig U, et al. (2021) Kinetic and Structural Characterization of the Self-Labeling Protein Tags HaloTag7, SNAP-tag, and CLIP-tag. Biochemistry 60:2560–2575.
